# Season of Conception and Risk of Cerebral Palsy

**DOI:** 10.1001/jamanetworkopen.2023.35164

**Published:** 2023-09-22

**Authors:** Haoran Zhuo, Beate Ritz, Joshua L. Warren, Zeyan Liew

**Affiliations:** 1 Department of Environmental Health Sciences, Yale School of Public Health, New Haven, Connecticut; 2 Yale Center for Perinatal, Pediatric, and Environmental Epidemiology, Yale School of Public Health, New Haven, Connecticut; 3 Department of Epidemiology, Fielding School of Public Health, University of California, Los Angeles; 4 Department of Neurology, School of Medicine, University of California, Los Angeles; 5 Department of Biostatistics, Yale School of Public Health, New Haven, Connecticut

## Abstract

**Importance:**

Cerebral palsy (CP) is the most prevalent neuromotor disability in childhood, but for most cases the etiology remains unexplained. Seasonal variation in the conception of CP may provide clues for their potential etiological risk factors that vary across seasons.

**Objective:**

To evaluate whether the month or season of conception is associated with CP occurrence.

**Design, Setting, and Participants:**

This statewide cohort study examined more than 4 million live births that were registered in the California birth records during 2007 to 2015 and were linked to CP diagnostic records (up to year 2021). Statistical analyses were conducted between March 2022 and January 2023.

**Exposures:**

The month and season of conception were estimated based on the child’s date of birth and the length of gestation recorded in the California birth records.

**Main Outcomes and Measures:**

CP status was ascertained from the diagnostic records obtained from the Department of Developmental Services in California. Poisson regression was used to estimate the relative risk (RR) and 95% CI for CP according to the month or the season of conception, adjusting for maternal- and neighborhood-level factors. Stratified analyses were conducted by child’s sex and neighborhood social vulnerability measures, and the mediating role of preterm birth was evaluated.

**Results:**

Records of 4 468 109 children (51.2% male; maternal age: 28.3% aged 19 to 25 years, 27.5% aged 26 to 30 years; maternal race and ethnicity: 5.6% African American or Black, 13.5% Asian, 49.8% Hispanic or Latinx of any race, and 28.3% non-Hispanic White) and 4697 with CP (55.1% male; maternal age: 28.3% aged 19 to 25 years, 26.0% aged 26 to 30 years; maternal race and ethnicity: 8.3% African American or Black, 8.6% Asian, 54.3% Hispanic or Latinx of any race, and 25.8% non-Hispanic White) were analyzed. Children conceived in winter (January to March) or spring (April to June) were associated with a 9% to 10% increased risk of CP (winter: RR, 1.09 [95% CI, 1.01-1.19]; spring: RR, 1.10 [95% CI, 1.02-1.20]) compared with summer (July to September) conceptions. Analyses for specific months showed similar results with children conceived in January, February, and May being at higher risk of CP. The associations were slightly stronger for mothers who lived in neighborhoods with a high social vulnerability index, but no child sex differences were observed. Only a small portion of the estimated association was mediated through preterm birth.

**Conclusions and Relevance:**

In this cohort study in California, children conceived in winter and spring had a small increase in CP risk. These findings suggest that seasonally varying environmental factors should be considered in the etiological research of CP.

## Key Points

**Question:**

Is the season at conception associated with the risk of childhood cerebral palsy?

**Findings:**

In this cohort study of more than 4 million births from California during 2007 to 2015, winter and spring conceptions had a 10% increased risk of cerebral palsy compared with summer, whereas no consistent differences were found for fall. The associations remained robust after adjustment for maternal individual- and community-level confounders.

**Meaning:**

This study’s results suggest that future etiological research on cerebral palsy should examine the effects of seasonally varying environmental factors.

## Teaser

This cohort study evaluates whether the month or season of conception is associated with cerebral palsy occurrence in California.

## Introduction

Cerebral palsy (CP) remains the most common neuromotor and physical disability that affects 2 to 3 children per 1000 live births in the US.^1^ CP disproportionally affects preterm children and male infants.^2,3,4,5^ The lifelong and comorbid health conditions of CP bring heavy burdens to patients, their households, and the health care system.^6,7^ However, etiological causes for most CP cases remain unknown.^8^

Epidemiological investigation of seasonal patterns of disease can provide clues about the role of seasonally varying environmental risk factors.^9,10^ For instance, studies have reported seasonal variations of preterm birth,^9^ a risk factor for CP,^8^ and other neurological disorders in childhood such as autism^10,11^; prenatal exposures that vary across seasons (ie, infection,^12,13,14^ air pollution,^15,16,17^ agricultural pesticides^18,19^) have been identified as risk factors of these health conditions. However, studies of seasonal variation of CP are limited, with only a small Polish case-control study reporting the increased CP cases from spring births.^20,21^ Here, we conducted a statewide cohort study of more than 4 million births from 2007 to 2015 in California to investigate whether CP diagnosis in childhood was associated with the month or season of conception as a marker for seasonally varying exposures that occurred in early gestation. Given the time of birth can be complicated by the length of gestation that has strong influences on CP risk, we assessed the season of conception as our main focus to capture exposure effects in the early pregnancy that shape the susceptibility of fetal brain injury,^14,18^ and we evaluated preterm birth as a potential mediating factor.

## Methods

This cohort study was approved by the official institutional review board at Yale University and the California Committee for the Protection of Human Subjects. This study was exempt from informed consent requirements as there was no contact with the study population. We followed the Strengthening the Reporting of Observational Studies in Epidemiology (STROBE) reporting guideline.

### Study Population

We created a statewide birth cohort that includes 4 652 013 live births registered in the California birth records during 2007 to 2015. We linked this birth cohort to the CP diagnostic records (up to year 2021) maintained by the California Department of Developmental Services (DDS) to ascertain CP cases. The DDS provides service and support to residents in California who have developmental disabilities or conditions (including CP) through 21 statewide regional centers.^22^ We used a probabilistic linkage algorithm to match the DDS records and the California birth records using parental and child’s identifying information (first and last name, date of birth, sex of child).^18,23,24^ CP cases registered in the DDS with a birthplace other than California were not eligible for the study. Overall, we identified a total of 4942 CP cases (linkage rate of 93%), with nonlinkages mainly due to missing identifying information.

We excluded birth records with missing information on maternal residential address or the date of last menstrual period, and records (<0.3%) with potential coding errors such as (1) having a length of gestation less than 20 weeks or greater than 50 weeks, and (2) birth weight less than 500 g or greater than 6000 g. The final sample included 4 468 109 live births and 4697 children with CP (eFigure 1 in Supplement 1).

### Outcomes

The presence of CP was ascertained from the DDS and defined as a group of nonprogressive lesions or disorders in the brain that are characterized by paralysis, spasticity, or abnormal movement and/or posture control that manifested in early childhood.^25^ We also extracted the motor dysfunction types and location of affected limbs of CP for analyses.

### Exposures and Covariates

Using the child’s date of birth and the length of gestation calculated from the last menstrual period in the birth records, we estimated the date of conception and determined the conceiving month and season. The season of conception was conceptualized as a proxy variable to investigate the overall association between various environmental factors and CP risk and was defined as winter (January to March), spring (April to June), summer (July to September), and fall (October to December).

We extracted maternal and child sociodemographic characteristics from the birth records and selected potential confounders that may influence pregnancy planning, fertility, and birth rates based on literature.^23,26,27^ Moreover, we obtained the census tract–level social vulnerability index (SVI) created by the US Centers for Disease Control and Prevention (CDC) and linked this measure to our geocoded maternal residential addresses from the birth records. The SVI ranks all census tracts in the US using a percentile ranking on 15 social factors, then the summation of all factors is taken, reordered among census tracts, and calculated to get the total score, where higher value indicates higher risk group.^28^ Additionally, CDC labels “flagged high vulnerability areas” as census tracts with any of the 15 factors ranked at the 90th percentile or above, and domain-specific high-risk group (socioeconomic status, household composition and disability, minority status and language, and housing type and transportation) based on factors within each domain.^28^ The SVI data for California were reported for years of 2010 and 2014 that overlapped with our study period. We assigned 2010 SVI data to the birth years 2007 to 2010, and 2014 SVI to birth years 2011 to 2015.

### Statistical Analysis

We used a generalized linear model of Poisson regression^29,30^ to estimate the relative risk (RR) and the model-based 95% CI for CP according to the month or season of conception, using July or summer as the reference groups that were similar to a previous study.^10^ In the primary model, we adjusted for birth year (continuous), maternal age at delivery (≤18 years, 19-25 years, 26-30 years, 31-35 years, >35 years), maternal self-reported race and ethnicity (African American or Black, Asian, Hispanic or Latinx of any race, non-Hispanic White, and others including Pacific Islander, American Indian, Eskimo, Aleut, and unspecified groups), educational attainment (<12th grade, high school or diploma, college, and above), prepregnancy body mass index (calculated as weight in kilograms divided by height in meters squared; <18.5, 18.5 to <25, 25 to <30, ≥30), smoking during pregnancy (yes or no), the total SVI (continuous), the DDS catchment area (categorical),^31^ and child’s sex (male or female). In sensitivity analyses, we further adjusted for maternal prenatal care (yes or no), source of payment for prenatal care (government, private, self-pay, others), receipt of the Special Supplemental Nutrition Program for Women, Infants, and Children (WIC) benefits (yes or no), and maternal parity (1, 2, ≥3). Maternal race and ethnicity were assessed because CP prevalence is known to vary by race and ethnicity subgroups in California. We also performed sensitivity analyses using singleton births to address concerns of higher CP prevalence among multiple births,^32^ and adjusted for the exact birth year to relax the linearity assumption of using a continuous birth year. As DDS does not provide the age of diagnosis, we conducted a sensitivity analysis by restricting to children with CP who were aged less than 6 years at the DDS services to assess the importance of the duration of study follow-up time.

CP is more common in male individuals,^4^ and we conducted stratified analyses by child’s sex to evaluate potential heterogeneity. Moreover, we evaluated the geographical differences by implementing stratifications on birth regions (northern, central, and southern California) (eFigure 2A in Supplement 1), and by rural and urban areas of census blocks (eFigure 2B in Supplement 1). We also evaluated potential differences across maternal individual or neighborhood socioeconomic status by stratifying on maternal race and ethnicity, education level, and SVI. We performed tests of heterogeneity by assessing *P* values of the product term between the season of conception and each of the potential effect modifiers in regression models with the significance threshold of 2-sided *P* < .05.

Furthermore, we investigated whether the results differ by main CP subphenotypes (eg, spastic, ataxic, dyskinetic, and others), and the limb involvement (unilateral or bilateral) among spastic CP. Preterm birth is a strong risk factor for CP and seasonal variation on preterm deliveries has been reported.^8^ Therefore, we performed mediation analysis under the counterfactual framework to examine whether preterm birth (defined as less than 37 weeks) mediated the overall associations between the season of conception and CP. We used the marginal structural model with inverse probability weighting method embedded in the CMAverse package in R^33^ to estimate the natural direct and indirect effects treating preterm as a mediator and allowed for potential exposure-mediator interactions.^34^ We also evaluated the mediating roles of neonatal Apgar score at 5 minutes and maternal preeclampsia in sensitivity analyses. Statistical analyses were conducted in SAS version 9.4 (SAS Institute) and R version 4.1.2. (R Project for Statistical Computing) from March 2022 to January 2023.

## Results

In this California cohort, records of 4 468 109 children (2 288 300 [51.2%] were male; maternal age: 1 265 393 [28.3%] aged 19 to 25 years, 1 227 157 [27.5%] aged 26 to 30 years; maternal race and ethnicity: 252 080 [5.6%] African American or Black, 601 781 [13.5%] Asian, 2 223 286 [49.8%] Hispanic or Latinx of any race, and 1 262 872 [28.3%] non-Hispanic White) and 4697 with CP (2586 [55.1%] were male; maternal age: 1328 [28.3%] aged 19 to 25 years, 1222 [26.0%] aged 26 to 30 years; maternal race and ethnicity: 392 [8.3%] African American or Black, 406 [8.6%] Asian, 2550 [54.3%] Hispanic or Latinx of any race, and 1210 [25.8%] non-Hispanic White) were analyzed. Children born to mothers who were identified as African American or Black and Hispanic or Latinx were more likely to have CP, so did children born to mothers who were older at delivery, were overweight or obese, with a lower education level, or smoked during pregnancy. As expected, CP disproportionally affected preterm birth and male children (Table 1). We estimated that children conceived in winter (RR, 1.09 [95% CI, 1.01-1.19]) or spring (RR, 1.10 [95% CI, 1.02-1.20]) had a 9% to 10% higher risk of CP compared with children conceived in summer. When studying the specific month of conception, a 15% higher risk of CP was observed among children who were conceived in January (RR, 1.15 [95% CI, 1.01-1.32]), February (RR, 1.15 [95% CI, 1.01-1.33]), or May (RR, 1.15 [95% CI, 1.01-1.33]) compared with July (Figure). These results remained consistent when we adjusted for additional maternal sociodemographic and health care–related factors or adjusted for the exact birth year (eTable 1 in Supplement 1) or limited to singleton births (eTable 2 in Supplement 1) or to children aged less than 6 years at the DDS services (eTable 3 in Supplement 1).

**Table 1.** Characteristics of the Study Population in California, 2007-2015

Characteristic	No. (%)^a^	
	Total live births (N = 4 468 109)	Children diagnosed with CP (n = 4697)
Maternal age at delivery, y		
≤18	198 286 (4.4)	260 (5.5)
19-25	1 265 393 (28.3)	1328 (28.3)
26-30	1 227 157 (27.5)	1222 (26.0)
31-35	1 114 772 (24.9)	1082 (23.0)
>35	662 472 (14.8)	805 (17.1)
Unknown	29 (<0.1)	0
Maternal race and ethnicity		
African American or Black	252 080 (5.6)	392 (8.3)
Asian	601 781 (13.5)	406 (8.6)
Hispanic or Latinx of any race	2 223 286 (49.8)	2550 (54.3)
Non-Hispanic White	1 262 872 (28.3)	1210 (25.8)
Other^b^	45 128 (1.0)	48 (1.0)
Unknown	82 962 (1.9)	91 (1.9)
Maternal education level		
<12th Grade	943 978 (21.1)	1169 (24.9)
High school or diploma	2 190 827 (49.0)	2452 (52.2)
College and above	1 169 746 (26.2)	911 (19.4)
Unknown	163 558 (3.7)	165 (3.5)
Maternal prepregnancy BMI		
<18.5	169 032 (3.8)	158 (3.4)
18.5 to <25.0	2 059 780 (46.1)	1849 (39.4)
25.0 to <30.0	1 081 206 (24.2)	1202 (25.6)
≥30	880 910 (19.7)	1156 (24.6)
Unknown	277 181 (6.2)	332 (7.1)
Maternal smoking during pregnancy		
No	4 373 950 (97.9)	4543 (96.7)
Yes	94 159 (2.1)	154 (3.3)
Year of birth		
2007-2010	2 052 652 (45.9)	2365 (50.4)
2011-2015	2 415 457 (54.1)	2332 (49.6)
Sex of children		
Male	2 288 300 (51.2)	2586 (55.1)
Female	2 179 809 (48.8)	2111 (44.9)
Preterm birth		
No	4 041 090 (90.4)	3073 (65.4)
Yes	427 019 (9.6)	1624 (34.6)
SVI of maternal residence at birth, mean (SD)^c^		
Total score	0.58 (0.28)	0.62 (0.27)
Socioeconomic domain	0.57 (0.29)	0.62 (0.28)
Household domain	0.56 (0.29)	0.60 (0.27)
Minority status domain^d^	0.58 (0.27)	0.62 (0.27)
Transportation domain	0.55 (0.28)	0.57 (0.28)
Unknown	180 (<0.1)	0

Abbreviations: BMI, body mass index (calculated as weight in kilograms divided by height in meters squared); SVI, social vulnerability index.

^a^ May not add to 100% due to rounding.

^b^ Other race and ethnicity included Pacific Islander, American Indian, Eskimo, Aleut, and other and/or unspecified.

^c^ SVI ranges from 0 to 1, with higher values indicating higher risk.

^d^ Minority status domain included race and ethnicity (not White) and language (speak English less than well).

**Figure.**
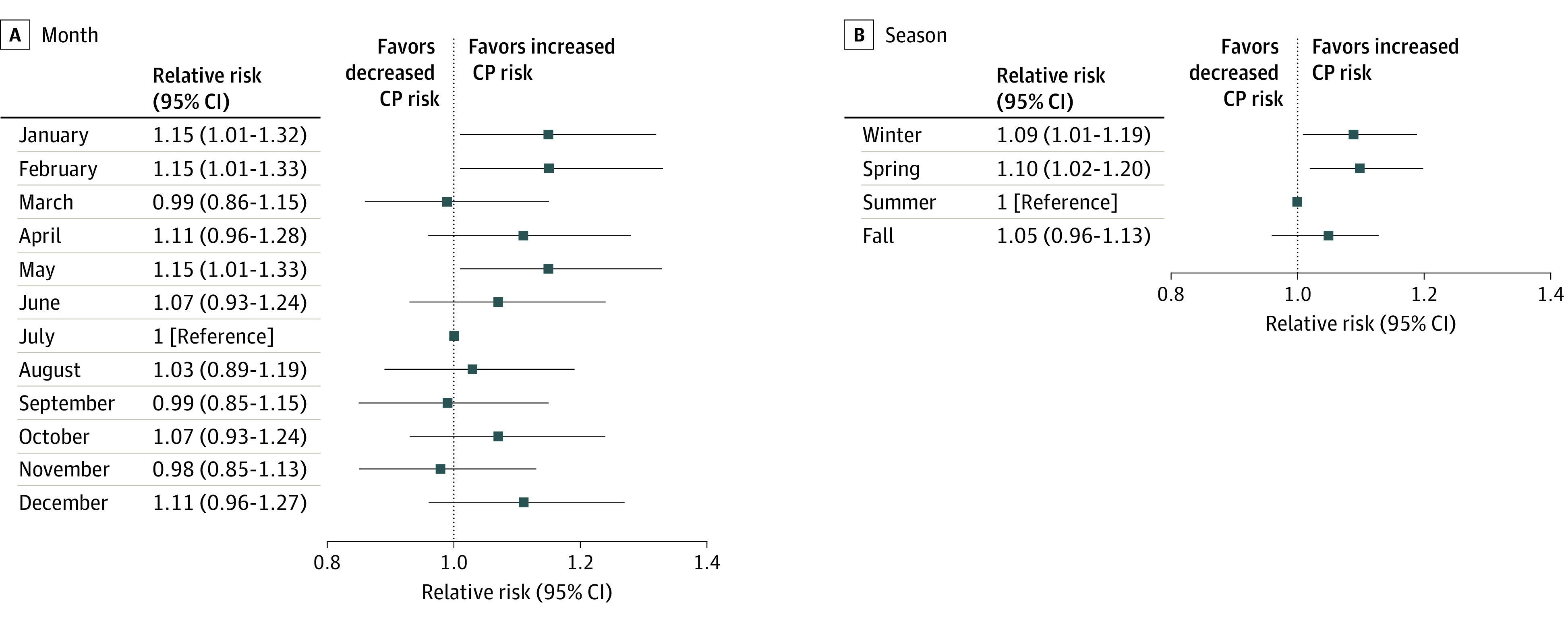
Month of Conception or Season of Conception and Cerebral Palsy (CP) Risk in California The risk ratio and 95% CI were estimated using generalized linear model adjusting for birth year, child’s sex, maternal age at delivery, race and ethnicity, education, smoking during pregnancy, prepregnancy body mass index (calculated as weight in kilograms divided by height in meters squared), maternal residential census tract–level social vulnerability index, and the Department of Developmental Service catchment area. The season of winter includes the months January to March, spring includes April to June, summer includes July to September, and fall includes October to December.

In stratified analyses, we estimated that the associations between winter and spring conceptions and CP risk were stronger if mothers resided in high-risk neighborhoods in terms of socioeconomic factors (winter: RR, 1.17 [95% CI, 1.01-1.35], spring: RR, 1.24 [95% CI, 1.07-1.43]) and racial and ethnic minority status (winter: RR, 1.18 [95% CI, 1.01-1.41], spring: RR, 1.23 [95% CI, 1.05-1.48]) (Table 2). The associations were also stronger among those with lower maternal education (eg, high school and below), but no consistent differences were among maternal individual race and ethnicity (eTable 4 in Supplement 1). Comparable associations were observed among male and female offspring (eTable 5 in Supplement 1). Winter months were slightly more strongly associated with CP risk among the northern and central regions of California, whereas spring associations were more robust among the central regions. Stronger effect estimates for spring and fall conceptions were observed in rural areas, although the estimated 95% CIs contained the null (eTable 6 in Supplement 1).

**Table 2.** Associations Between the Season of Conception and CP Risk in California, 2007-2015, Stratified by Maternal Neighborhood SVI

Seasons of conception	CP cases	Total births	Risk ratio (95% CI)^a^	CP cases	Total births	Risk ratio (95% CI)^a^	*P* for interaction
**Neighborhoods with high vulnerability (rank ≥90th)**			**Neighborhoods with a lower vulnerability (rank <90th)**		
**Total SVI**							
Winter	739	636 045	1.13 (1.02-1.26)	429	462 211	1.04 (0.91-1.18)	.18
Spring	757	627 450	1.16 (1.04-1.28)	440	460 147	1.02 (0.90-1.17)	.14
Summer	647	621 979	1 [Reference]	437	468 089	1 [Reference]	NA
Fall	768	689 069	1.07 (0.96-1.18)	480	502 939	1.02 (0.89-1.16)	.55
**Subdomain: socioeconomic status**							
Winter	413	301 887	1.17 (1.01-1.35)	755	796 369	1.05 (0.95-1.17)	.14
Spring	433	297 109	1.24 (1.07-1.43)	764	790 488	1.04 (0.94-1.15)	.04
Summer	341	290 025	1 [Reference]	743	800 043	1 [Reference]	NA
Fall	405	328 002	1.05 (0.91-1.21)	843	864 006	1.04 (0.95-1.15)	.96
**Subdomain: racial and ethnic minority status**							
Winter	285	210 387	1.18 (1.01-1.41)	883	887 904	1.07 (0.97-1.17)	.15
Spring	294	207 569	1.23 (1.05-1.48)	903	880 060	1.06 (0.97-1.17)	.06
Summer	229	201 290	1 [Reference]	855	888 817	1 [Reference]	NA
Fall	298	227 825	1.15 (0.97-1.37)	950	964 220	1.02 (0.93-1.12)	.22
**Subdomain: household composition**							
Winter	392	329 239	1.08 (0.93-1.24)	776	769 052	1.10 (0.99-1.23)	.89
Spring	422	324 537	1.17 (1.02-1.35)	775	763 093	1.07 (0.97-1.18)	.27
Summer	356	322 286	1 [Reference]	728	767 821	1 [Reference]	NA
Fall	408	358 185	1.03 (0.89-1.18)	840	833 860	1.06 (0.96-1.17)	.76
**Subdomain: transportation-related factors**							
Winter	495	421 776	1.11 (0.99-1.27)	673	676 515	1.08 (0.97-1.20)	.53
Spring	525	416 466	1.18 (1.04-1.34)	672	671 163	1.05 (0.94-1.17)	.16
Summer	438	411 189	1 [Reference]	646	678 918	1 [Reference]	NA
Fall	528	456 207	1.08 (0.95-1.23)	720	735 838	1.02 (0.92-1.14)	.50

Abbreviations: CP, cerebral palsy; SVI, social vulnerability index; NA, not applicable.

^a^ Adjusted for birth year, child’s sex, maternal age at delivery, race and ethnicity, education, smoking during pregnancy, and prepregnancy body mass index.

The main results were slightly stronger for the spastic CP subtype (winter: RR, 1.16 [95% CI, 1.03-1.30], spring: RR, 1.18 [95% CI, 1.05-1.38]) (eTable 7 in Supplement 1). Small estimated indirect effects were observed for winter (indirect RR, 1.01, [95% CI, 1.00-1.01]; mediated percentage, 7.45%) and spring (indirect RR, 1.01, [95% CI, 1.00-1.01]; mediated percentage, 6.95%) conceptions and CP (Table 3). We also found a small estimated indirect effect from the 5-minute Apgar score for spring (mediated percentage, 10.4%) but not winter (mediated percentage, 0.8%) conceptions, while the estimated mediating effects from preeclampsia were close to null (winter: mediated percentage, 0.4%; spring: mediated percentage, 0.9%) (eTable 8 in Supplement 1).

**Table 3.** Mediation Analysis of the Associations Between the Season of Conception and Cerebral Palsy Risk With Preterm Birth as a Mediating Factor

Season of conception	Natural direct effect, risk ratio (95% CI)^a^	Natural indirect effect, risk ratio (95% CI)^a^	Percentage mediated, %
Winter	1.09 (1.01-1.18)	1.01 (1.00-1.01)	7.45
Spring	1.10 (1.01-1.19)	1.01 (1.00-1.01)	6.95
Summer	1 [Reference]	1 [Reference]	1 [Reference]
Fall	1.05 (0.75-1.33)	0.99 (0.62-1.14)	NA

Abbreviation: NA, not applicable.

^a^ Adjusted for birth year, child’s sex, maternal age at delivery, race/ethnicity, education, smoking during pregnancy, and prepregnancy body mass index (calculated as weight in kilograms divided by height in meters squared), and maternal residential census tract–level social vulnerability index.

## Discussion

We observed that winter and spring conceptions were associated with a small increase of CP risk compared with summer conceptions in California, after adjusting for a range of individual and neighborhood factors. The risk increases were stronger in subpopulations who lived in areas of higher social risk or among mothers with lower education. Preterm birth only explained a small proportion of these seasonal differences. Overall, the estimated increased risk of CP across the season of conceptions remained small, thus our results do not support a change in family planning.

Investigating seasonal variations in disease occurrence can provide clues about etiologically relevant factors. To our knowledge, only a prior case-control study in Poland during years of 1990 to 1999 and 2000 to 2014 has reported a higher CP incidence for spring births.^20,21^ However, this study did not adjust for any confounding factors and findings were from late pregnancy exposures and can be distinct from exposures in early pregnancy. Previously, higher risk of autism was observed among children conceived in winter compared with those conceived in summer in a California cohort (1990-2002) and a Scottish cohort (2006-2011).^10,35^ Moreover, the Scottish cohort also reported highest risks of intellectual disabilities and learning difficulties among children conceived in winter and the lowest in summer.^35^ Results for seasonally increased risks may differ in studies that assessed season of conception vs season at birth as these would cover different susceptible windows for exposures in pregnancy.

It is possible that multiple seasonally varying factors contribute to the seasonality of CP. First, maternal infections during pregnancy, especially when accompanied by fever or an increase in cytokines, have been frequently linked to CP given that inflammation may cause fetal brain damage.^36,37,38^ In particular, maternal bacterial infections such as chorioamnionitis and urinary tract infections (UTIs) in late pregnancy have been linked to CP in the offspring.^14,39,40,41^ However, there is little evidence on the seasonal pattern of chorioamnionitis and therefore may contribute little to the seasonal pattern of CP. Instead, warmer weather (especially summer months) has been reported to increase the risk for UTIs among women in a dose-response relationship with a plateau by 35 °C.^42,43^ Winter conceptions that have later stages of pregnancy in summer and fall would therefore be susceptible to UTI infections.^42,43^ On the other hand, viral infections generally have winter peaks, such as influenza, chicken pox, and rubella.^44^ However, the prevalence of both chicken pox and rubella is low in the US due to effective vaccination programs.^45^ Instead, the influenza infection annual peak of December to February in the US^46^ clearly overlaps with winter conceptions where we found an increased risk of CP. Early gestational exposures to influenza have also been associated with delays in psychomotor development^47^ and autism^12^ in the US, but not yet with CP.

Pesticides exposures are prevalent in California, and our previous study conducted in agricultural regions of California reported that maternal first-trimester exposures to pesticide compounds that disrupt the endocrine systems were associated with CP risk.^18^ In fact, more than 150 million agricultural pesticide active ingredients are applied every year in California with a major peak application from May to July,^48^ a period that overlaps with the first or second pregnancy trimesters for conceptions in late winter and spring. In addition, agricultural pesticides were most heavily applied in the central regions of California, where we also observed a stronger effect size of increased risk of CP. Pesticides can evaporate and drift from the point of application and further contaminate indoor environments and expose nearby residents.^49^ In addition, exposure to air pollution during pregnancy (such as fine particulate matters) can negatively affect neurodevelopment^15,16^ and motor function in the offspring.^50,51^ Particles have seasonal peaks in late fall and winter with strong seasonal contrasts were seen especially in northern and central regions of California.^52^ Currently, epidemiological evidence for associations between ambient air pollution and CP is lacking and requires further investigation.

Prenatal exposures to extreme temperature, especially for heat exposure effects in late pregnancy, have been linked to adverse birth outcomes,^53,54^ but whether the exposure effects also influence fetal brain development has not been well studied and requires further investigation. It is also possible for some protective factors that occur in the summer and the start of pregnancy as an alternative explanation to our findings. One potential protective environmental factor in summer could be the sunlight exposure and its correlated serum vitamin D levels,^55,56,57,58^ which might play a limited role in the southern regions of California with abundant sunlight exposures throughout the year.^59^ To our knowledge, no other protective factors have been discussed in previous literature,^9,10,11^ but this possibility cannot be ruled out.

In California, CP is more prevalent among certain racial and ethnic minority and socially at-risk subgroups.^23^ We found that mothers living in neighborhoods with higher socioeconomic risk were more strongly affected by season. This may suggest higher rates of infections from crowded housing conditions or higher exposures to seasonal environmental pollutants that disproportionately affect low-income communities.^60,61^ Moreover, low individual-level socioeconomic status may limit the risk mitigation behaviors and options that would reduce environmental exposures such as avoiding outdoor activities during hot seasons or periods of high air pollution or pesticide exposures, as well as having access to indoor air conditioning that reduces effects from extremely hot weather.^62^ Only a weak mediating effect via preterm birth was found and may be due to the fact that a variety of seasonally varying factors contribute to CP but not necessarily to preterm birth.^63^ We did not find consistent mediating effects for neonatal Apgar scores and maternal preeclampsia.

### Strengths and Limitations

This is a large cohort study of 4 million live births in California and our CP cases were ascertained from the DDS records, limiting recall bias and self-selection participation bias. We were able to evaluate subgroup differences by geospatial locations, neighborhood social vulnerability, and adjusted for a range of potential confounders and quantified the potential mediating effect of preterm birth.

Our study also has limitations. First, there is a possibility of outcome misclassification given that CP cases with mild symptoms less in need of services might not be captured by DDS,^23^ so did cases diagnosed outside of California. However, only a very small portion of such cases would be included among all births, and this misclassification is likely to be nondifferential by season of conception. Because of this underascertainment and the lack of first diagnosed age of CP cases in the DDS, we were unable to directly compute and compare the incidence rate measures. Second, our results might also be affected by the potential misclassification from estimated month of conceptions using last menstrual.^64^ However, a validation study suggested that the misclassification of gestational week is usually limited to 2 to 4 weeks for most records,^65,66^ and it is reassuring that we observed consistent patterns for most of the consecutive months. While family characteristics such as pregnancy planning that may vary by season were not evaluated,^67^ our study considered a number of individual-level and community-level confounding factors, and we are not aware of other strong confounding factors. Stratified analyses have lower statistical precision and a higher risk for chance errors when analyzing smaller subgroups. The mediation analysis relied on an untestable assumption that there was no uncontrolled confounding between the exposure, the mediator, or the outcome.^34^ Additionally, the time of conception is a contextual variable that represents various seasonally varying factors, which can have different strengths, directions, and critical windows on the associations with CP risk.

## Conclusions

In this cohort study in California, we found that winter and spring conceptions had a small increased risk of CP. To our knowledge, this was the largest cohort study to date to suggest that seasonally varying factors may contribute to CP occurrence, and the strength of observed associations may also be affected by social vulnerability. While the results should not be used to promote a change in family planning, our findings suggested that future etiological research on CP should investigate the exposure effects from seasonally varying environmental risk factors.

## Notes

Supplement 1. **eFigure 1.** Flowchart of the Study Population **eFigure 2.** Birth Regions, DDS Center Locations, and Urban Areas of California **eTable 1.** Sensitivity Analysis With Additional Adjustment for More Covariates **eTable 2.** Sensitivity Analysis on Live Singleton Births Only **eTable 3.** Sensitivity Analysis on Children Aged Less Than 6 at the DDS Services Establishment **eTable 4.** Stratified Analysis by Maternal Individual SES (Race, Education) **eTable 5.** Stratified Analysis by Sex **eTable 6.** Stratified Analysis by Region of Birth Counties **eTable 7.** Stratified Analysis by Subtypes of CP and Locations of Spastic CP **eTable 8.** Mediation Analysis with Apgar Score and Maternal Pre-Eclampsia as Mediating Factors

Supplement 2.
Data Sharing Statement
