# Supplementary material for: Season of Conception and Risk of Cerebral Palsy
Source: JAMA Netw Open. 2023 Sep 22;6(9):e2335164. doi: 10.1001/jamanetworkopen.2023.35164 (PMC10517373; doi:10.1001/jamanetworkopen.2023.35164)
Supplement: Supplement 1. — eFigure 1. Flowchart of the Study Population eFigure 2. Birth Regions, DDS Center Locations, and Urban Areas of California eTable 1. Sensitivity Analysis With Additional Adjustment for More Covariates eTable 2. Sensitivity Analysis on Live Singleton Births Only eTable 3. Sensitivity Analysis on Children Aged Less Than 6 at the DDS Services Establishment eTable 4. Stratified Analysis by Maternal Individual SES (Race, Education) eTable 5. Stratified Analysis by Sex eTable 6. Stratified Analysis by Region of Birth Counties eTable 7. Stratified Analysis by Subtypes of CP and Locations of Spastic CP eTable 8. Mediation Analysis with Apgar Score and Maternal Pre-Eclampsia as Mediating Factors [file jamanetwopen-e2335164-s001.pdf]

Supplemental Online Content

Zhuo H, Ritz B, Warren JL, Liew Z. Season of conception and risk of cerebral palsy. *JAMA Netw Open*. 2023;6(9):e2335164. doi:10.1001/jamanetworkopen.2023.35164

eFigure 1. Flowchart of the Study Population

eFigure 2. Birth Regions, DDS Center Locations, and Urban Areas of California

eTable 1. Sensitivity Analysis With Additional Adjustment for More Covariates

eTable 2. Sensitivity Analysis on Live Singleton Births Only

eTable 3. Sensitivity Analysis on Children Aged Less Than 6 at the DDS Services Establishment

eTable 4. Stratified Analysis by Maternal Individual SES (Race, Education)

eTable 5. Stratified Analysis by Sex

eTable 6. Stratified Analysis by Region of Birth Counties

eTable 7. Stratified Analysis by Subtypes of CP and Locations of Spastic CP

eTable 8. Mediation Analysis with Apgar Score and Maternal Pre-Eclampsia as Mediating Factors

This supplemental material has been provided by the authors to give readers additional information about their work.

© 2023 Zhuo H et al. *JAMA Network Open.*

eFigure 1. Flowchart of the study population

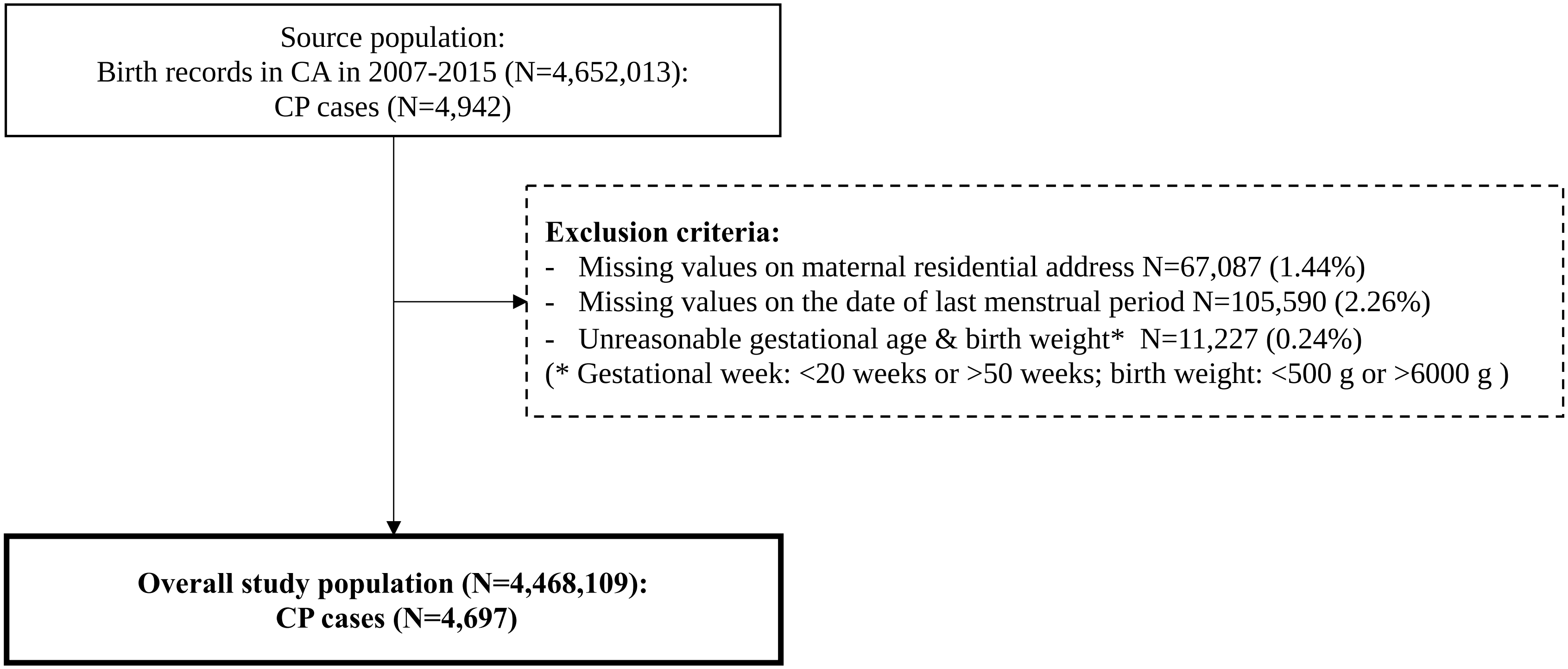

© 2023 Zhuo H et al. *JAMA Network Open.*

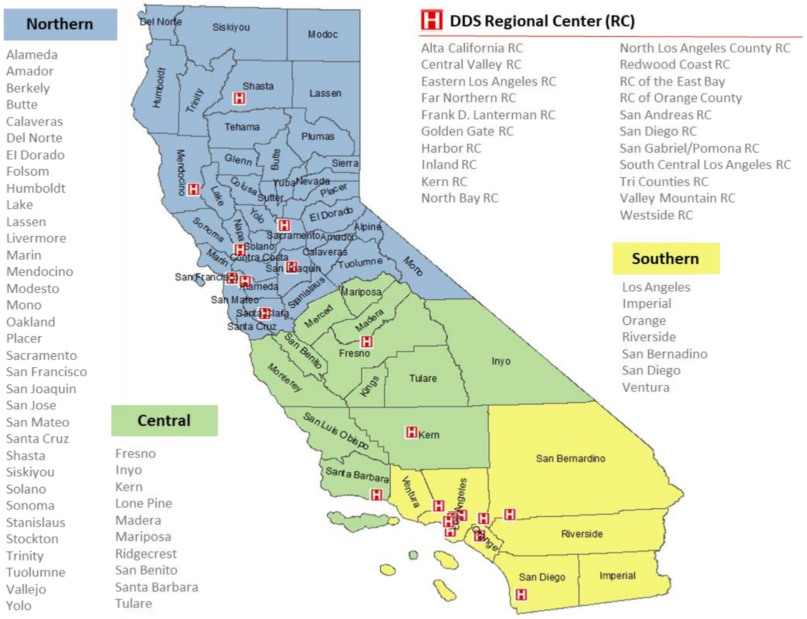

eFigure 2a. Birth regions and DDS center locations

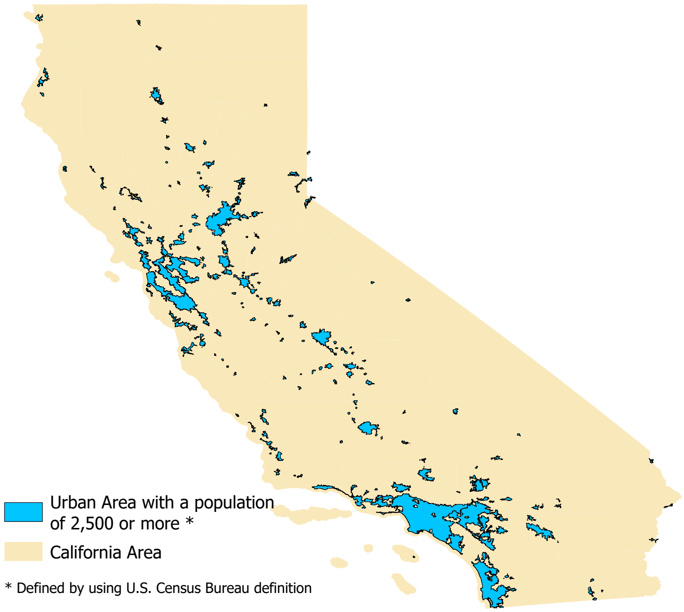

eFigure 2b. Urban area in California

© 2023 Zhuo H et al. *JAMA Network Open.*

eTable 1. Associations between the month or the season of conception and cerebral palsy (CP) risk in California, 2007-2015, adjusting for additional covariates

Conception months/seasons	CP cases	Total Births	Crude RR (95% CI)	RR (95% CI)[1]	RR (95% CI)[2]
January	420	379,904	1.14 (1.00, 1.31)	1.14 (1.00, 1.32)	1.12 (0.98, 1.29)
February	396	354,325	1.14 (1.00, 1.32)	1.15 (1.00, 1.32)	1.13 (0.98 1.30)
March	352	364,071	0.98 (0.84, 1.13)	0.99 (0.85, 1.15)	0.97 (0.84, 1.13)
April	393	358,236	1.13 (0.98, 1.30)	1.10 (0.95, 1.27)	1.10 (0.95, 1.27)
May	419	367,340	1.16 (1.01, 1.33)	1.15 (1.00, 1.32)	1.15 (1.00, 1.32)
June	385	362,071	1.09 (0.94, 1.26)	1.07 (0.93, 1.24)	1.07 (0.93, 1.24)
July	354	359,058	Ref	Ref	Ref
August	374	367,543	1.04 (0.90, 1.20)	1.03 (0.89, 1.19)	1.03 (0.89, 1.19)
September	356	363,508	0.99 (0.86, 1.15)	0.99 (0.85, 1.14)	0.99 (0.86, 1.15)
October	416	390,509	1.07 (0.93, 1.23)	1.07 (0.93, 1.23)	1.07 (0.93, 1.23)
November	381	391,334	1.00 (0.86, 1.15)	0.98 (0.84, 1.13)	0.98 (0.84, 1.13)
December	451	410,210	1.11 (0.96, 1.27)	1.10 (0.96, 1.27)	1.10 (0.96, 1.27)
Winter (Jan-March)	1168	1,098,300	1.08 (1.00, 1.16)	1.09 (1.00, 1.18)	1.08 (0.98, 1.16)
Spring (April-June)	1197	1,087,647	1.11 (1.02, 1,20)	1.10 (1.01, 1.19)	1.10 (1.01, 1.19)
Summer (July-Sept)	1084	1,090,109	Ref	Ref	Ref
Fall (Oct-Dec)	1248	1,192,053	1.05 (0.97, 1.14)	1.04 (0.96, 1.13)	1.04 (0.96, 1.13)

[1]Adjusted for continuous birth year, child's sex, maternal age at delivery, race/ethnicity, education, smoking during pregnancy, pre-pregnancy BMI, maternal residential census-tract level social vulnerability index (SVI), and DDS catchment area. With additional adjustment for prenatal care, payment source for prenatal care, WIC benefits, and parity.

[2] Adjusted for categorical birth year (with 2005 as the reference), child's sex, maternal age at delivery, race/ethnicity, education, smoking during pregnancy, pre-pregnancy BMI, maternal residential census-tract level social vulnerability index (SVI), and DDS catchment area.

© 2023 Zhuo H et al. *JAMA Network Open.*

eTable 2. Associations between the month or the season of conception and cerebral palsy (CP) risk among singleton live births in California, 2007-2015

Conception months/seasons	CP cases	Total Births	RR (95% CI)
		**Month**	
January	328	367,472	1.11 (0.96, 1.28)
February	373	343,110	1.15 (1.00, 1.33)
March	324	352,575	0.97 (0.83, 1.13)
April	357	346,759	1.06 (0.92, 1.24)
May	383	355,948	1.12 (0.97, 1.29)
June	361	350,892	1.07 (0.92, 1.24)
July	332	347,688	Ref
August	349	356,252	1.03 (0.88, 1.19)
September	322	352,086	0.96 (0.82, 1.11)
October	385	378,253	1.06 (0.91, 1.22)
November	354	379,076	0.96 (0.83, 1.12)
December	414	398,586	1.07 (0.93, 1.24)
		**Season**	
Winter (Jan-March)	1079	1,063,157	1.08 (1.00, 1.18)
Spring (April-June)	1101	1,053,599	1.09 (1.01, 1.19)
Summer (July-Sept)	1003	1,056,026	Ref
Fall (Oct-Dec)	1153	1,155,915	1.04 (0.95, 1.13)

Adjusted for birth year, child's sex, maternal age at delivery, race/ethnicity, education, smoking during pregnancy, pre-pregnancy BMI, maternal residential census-tract level social vulnerability index (SVI), and DDS catchment area.

© 2023 Zhuo H et al. *JAMA Network Open.*

eTable 3. Associations between the season of conception and cerebral palsy (CP) risk in California among children with age less than 6 at DDS services establishment, 2007-2015

Conception Season	# Of CP	Total births	Adjusted RR	(95% CI)
Winter	244	1097376	1.25	(1.02, 1.53)
Spring	182	1086632	1.15	(0.93, 1.42)
Summer	155	1089180	Ref	
Fall	226	1191031	1.32	(1.08, 1.62)

Adjusted for child's sex, maternal age at delivery, race/ethnicity, education, smoking during pregnancy, pre-pregnancy BMI, and maternal residential census-tract level social vulnerability index (SVI).

© 2023 Zhuo H et al. *JAMA Network Open.*

eTable 4. Associations between the season of conception and cerebral palsy (CP) risk in California, 2007-2015, stratified by maternal education level and race/ethnicity

Season	CP/ Total births	RR (95% CI)	CP/ Total births	RR (95% CI)	CP/ Total births	RR (95% CI)	CP/ Total births	RR (95% CI)
**Maternal education level**								
	**High school and below**		**College and above**					
Winter	896/772764	1.10 (1.01, 1.21)	229/284114	1.04 (0.87, 1.25)				
Spring	949/765085	1.15 (1.05, 1.27)	206/283132	0.91 (0.76, 1.10)				
Summer	811/754320	Ref	238/296543	Ref				
Fall	965/842636	1.06 (0.97, 1.17)	238/305957	0.97 (0.81, 1.16)				
**Maternal race/ethnicity**								
	**Non-Hispanic White**		**Hispanic/Latinx of any race**		**African American/Black**		**Asian, and Other***	
Winter	309/301995	1.18 (1.01, 1.38)	622/548758	1.06 (0.95, 1.19)	96/62663	0.96 (0.72, 1.28)	117/164064	1.08 (0.83, 1.40)
Spring	295/306886	1.06 (0.90, 1.24)	660/540366	1.13 (1.01, 1.26)	105/61792	1.08 (0.82, 1.43)	112/158455	1.02 (0.78, 1.33)
Summer	290/319956	Ref	576/532767	Ref	94/60066	Ref	109/157266	Ref
Fall	316/334035	1.04 (0.89, 1.22)	692/601395	1.06 (0.95, 1.19)	97/67559	0.92 (0.69, 1.22)	116/167124	1.00 (0.77, 1.29)

Stratified by education, adjusted for birth year, child's sex, maternal age at delivery, race/ethnicity, smoking during pregnancy, pre-pregnancy BMI, maternal residential census-tract level social vulnerability index (SVI).
Stratified by race/ethnicity, adjusted for birth year, child's sex, maternal age at delivery, education, smoking during pregnancy, pre-pregnancy BMI, maternal residential census-tract level social vulnerability index (SVI).
*Other include Pacific Islander, American Indian, Eskimo, Aleut, and other-unspecified

P-value for interactions: P-value=0.03 for interaction term education level & Spring; all other p-values >0.10

© 2023 Zhuo H et al. *JAMA Network Open.*

eTable 5. Association between the season of conception and cerebral palsy (CP) risk in California, stratified by child's sex

Season	CP	Total births	RR	(95% CI)	CP	Total births	RR	(95% CI)	P-interaction
		Males				Females			
Winter	644	561626	1.09	(0.97, 1.21)	524	536670	1.10	(0.97, 1.24)	0.94
Spring	651	556053	1.08	(0.97, 1.21)	546	531593	1.13	(1.00, 1.27)	0.66
Summer	602	559647	1.00		482	530459	1.00		
Fall	689	610961	1.04	(0.93, 1.16)	559	581087	1.05	(0.93, 1.18)	0.92

Adjusted for birth year, maternal age at delivery, race/ethnicity, education, smoking during pregnancy, pre-pregnancy BMI, maternal residential census-tract level social vulnerability index (SVI).

© 2023 Zhuo H et al. *JAMA Network Open.*

eTable 6. Association between the season of conception and cerebral palsy (CP) risk in California, 2007-2015, stratified by geographical regions.

Season	CP	Total births	RR (95% CI)	CP	Total births	RR (95% CI)	CP	Total births	RR (95% CI)
		**Northern**			**Central**			**Southern**	
Winter	281	332078	1.20 (1.01, 1.42)	114	127936	1.34 (1.02, 1.77)	773	638286	1.02 (0.92, 1.13)
Spring	262	331601	1.06 (0.89, 1.26)	104	127498	1.16 (0.90, 1.52)	831	628548	1.10 (1.00, 1.22)
Summer	254	340936		91	127937		739	621236	
Fall	288	361979	1.07 (0.90, 1.26)	107	140812	1.06 (0.80, 1.40)	853	689262	1.03 (0.94, 1.14)
		**Urban**			**Rural**				
Winter	1127	1051594	1.09 (1.01, 1.19)	41	46706	1.11 (0.71, 1.74)			
Spring	1144	1041090	1.09 (1.01, 1.19)	53	46557	1.43 (0.94, 2.18)			
Summer	1043	1042891	Ref	41	47218	Ref			
Fall	1194	1140876	1.04 (0.96 1.13)	54	51177	1.24 (0.81, 1.90)			

Adjusted for birth year, maternal age at delivery, race/ethnicity, education, smoking during pregnancy, pre-pregnancy BMI, maternal residential census-tract level social vulnerability index (SVI).

P-value for interactions: All p-values >0.1

© 2023 Zhuo H et al. *JAMA Network Open.*

eTable 7. Associations between seasons of conception and cerebral palsy (CP) risk among stratified population by CP subtypes and locations of spastic CP in California, 2007-2015

Seasons of conception	CP cases	Total births	RR (95% CI)
**Spastic CP**			
Winter	596	1097728	1.16 (1.03, 1.30)
Spring	619	1087069	1.18 (1.05, 1.38)
Summer	522	1089547	Ref
Fall	640	1191445	1.11 (0.99, 1.25)
**Ataxic CP**			
Winter	55	1097187	1.04 (0.72, 1.53)
Spring	48	1086498	0.89 (0.60, 1.31)
Summer	54	1089079	Ref
Fall	57	1190862	0.97 (0.67, 1.40)
**Dyskinetic CP**			
Winter	23	1097155	0.92 (0.52, 1.61)
Spring	22	1086472	0.85 (0.48, 1.50)
Summer	26	1089051	Ref
Fall	30	1190835	1.04 (0.62, 1.76)
**Other subtypes (including Hypotonic and mixed CP)**			
Winter	484	1097616	1.04 (0.91, 1.18)
Spring	495	1086945	1.06 (0.93, 1.20)
Summer	466	1089491	Ref
Fall	502	1191307	0.98 (0.86, 1.11)
**Bilateral, Spastic CP**			
Winter	438	1097570	1.17 (1.01, 1.34)
Spring	447	1086897	1.17 (1.02, 1.34)
Summer	382	1089407	Ref
Fall	471	1191276	1.12 (0.98, 1.28)
**Unilateral, Spastic CP**			
Winter	136	1097268	1.14 (0.89, 1.45)
Spring	149	1086599	1.23 (0.97, 1.57)
Summer	121	1089146	Ref
Fall	149	1190954	1.12 (0.88, 1.42)

Adjusted for birth year, maternal age at delivery, race/ethnicity, education, smoking during pregnancy, pre-pregnancy BMI, maternal residential census-tract level social vulnerability index (SVI).

© 2023 Zhuo H et al. *JAMA Network Open.*

eTable 8. Mediation analysis of the associations between the season of conception and cerebral palsy (CP) risk with Apgar score and maternal pre-eclampsia as mediating factors

Season of conception	Natural direct effect risk ratio (95% CI)	Natural Indirect effect risk ratio (95% CI)	Percentage (%) Mediated
**Apgar score at 5 min**			
Winter	1.10 (1.04, 1.17)	1.00 (1.00, 1.01)	0.8%
Spring	1.10 (1.07, 1.16)	1.01 (1.01, 1.02)	10.4%
Summer	Ref	Ref	Ref
Fall	1.05 (0.98, 1.14)	1.00 (1.00, 1.01)	6.1%
**Maternal pre-eclampsia***			
Winter	1.12 (0.98, 1.21)	1.00 (1.00, 1.01)	0.4%
Spring	1.04 (0.99, 1.09)	1.00 (1.00, 1.01)	0.9%
Summer	Ref	Ref	Ref
Fall	1.04 (0.95, 1.20)	1.00 (1.00, 1.01)	4.0%

Adjusted for birth year, child's sex, maternal age at delivery, race/ethnicity, education, smoking during pregnancy, and pre-pregnancy BMI, and maternal residential census-tract level social vulnearity index (SVI).

*35% population has missing values on pre-eclampsia and the estimated total effects are slightly different from other total effects

© 2023 Zhuo H et al. *JAMA Network Open.*
