# Supplementary material for: Season of Conception and Risk of Cerebral Palsy
Source: JAMA Netw Open. 2023 Sep 22;6(9):e2335164. doi: 10.1001/jamanetworkopen.2023.35164 (PMC10517373; doi:10.1001/jamanetworkopen.2023.35164)
Supplement: Supplement 2. — Data Sharing Statement [file jamanetwopen-e2335164-s002.pdf]

Data Sharing Statement

Zhuo. Season of Conception and Risk of Cerebral Palsy. *JAMA Netw Open*. Published September 22, 2023. doi:10.1001/jamanetworkopen.2023.35164

Data
Data available: No
